# Outcomes of antiretroviral treatment programmes in rural Lesotho: health centres and hospitals compared

**DOI:** 10.7448/IAS.16.1.18616

**Published:** 2013-11-21

**Authors:** Niklaus Daniel Labhardt, Olivia Keiser, Motlalepula Sello, Thabo Ishmael Lejone, Karolin Pfeiffer, Mary-Ann Davies, Matthias Egger, Jochen Ehmer, Gilles Wandeler, the SolidarMed ART Program and IeDEA-Southern Africa

**Affiliations:** 1 SolidarMed Lesotho, Maseru, Lesotho; 2 University Medical Clinic, Kantonsspital Baselland, Liestal, Switzerland; 3 Institute of Social and Preventive Medicine, University of Bern, Bern, Switzerland; 4 SolidarMed Switzerland, Lucerne, Switzerland; 5 School of Public Health and Family Medicine, University of Cape Town, South Africa; 6 Department of Infectious Diseases, University Hospital Bern, Bern, Switzerland; 7 Department of Infectious Diseases, University of Dakar, Dakar, Senegal

**Keywords:** antiretroviral treatment, decentralization, rural Southern Africa, retention in care, task shifting, nurse-based care, HIV

## Abstract

**Introduction:**

Lesotho was among the first countries to adopt decentralization of care from hospitals to nurse-led health centres (HCs) to scale up the provision of antiretroviral therapy (ART). We compared outcomes between patients who started ART at HCs and hospitals in two rural catchment areas in Lesotho.

**Methods:**

The two catchment areas comprise two hospitals and 12 HCs. Patients ≥16 years starting ART at a hospital or HC between 2008 and 2011 were included. Loss to follow-up (LTFU) was defined as not returning to the facility for ≥180 days after the last visit, no follow-up (no FUP) as not returning after starting ART, and retention in care as alive and on ART at the facility. The data were analysed using logistic regression, competing risk regression and Kaplan-Meier methods. Multivariable analyses were adjusted for sex, age, CD4 cell count, World Health Organization stage, catchment area and type of ART. All analyses were stratified by gender.

**Results:**

Of 3747 patients, 2042 (54.5%) started ART at HCs. Both women and men at hospitals had more advanced clinical and immunological stages of disease than those at HCs. Over 5445 patient-years, 420 died and 475 were LTFU. Kaplan-Meier estimates for three-year retention were 68.7 and 69.7% at HCs and hospitals, respectively, among women (*p*=0.81) and 68.8% at HCs versus 54.7% at hospitals among men (*p*<0.001). These findings persisted in adjusted analyses, with similar retention at HCs and hospitals among women (odds ratio (OR): 0.89, 95% confidence interval (CI): 0.73–1.09) and higher retention at HCs among men (OR: 1.53, 95% CI: 1.20–1.96). The latter result was mainly driven by a lower proportion of patients LTFU at HCs (OR: 0.68, 95% CI: 0.51–0.93).

**Conclusions:**

In rural Lesotho, overall retention in care did not differ significantly between nurse-led HCs and hospitals. However, men seemed to benefit most from starting ART at HCs, as they were more likely to remain in care in these facilities compared to hospitals.

## Introduction

Scarce human resources for health are a major obstacle to the scale-up of antiretroviral therapy (ART) in rural Africa [1,2]. In response, the World Health Organization (WHO) recommends that, whenever possible, tasks should be shifted to less specialized health workers [3], in line with the WHO's public health approach to ART in resource-limited settings [4]. Nurses may partly or completely take over the provision of ART to HIV-infected patients [5]. To compensate for the additional workload, other tasks, such as HIV testing and counselling or adherence and psychosocial counselling, may be shifted to lay personnel. Such task shifting allows the decentralization of ART provision to nurse-led primary healthcare clinics.

Through task shifting and decentralization, several countries in sub-Saharan Africa, such as Zambia, Ethiopia and Malawi, managed to scale up ART provision substantially [6–8]. A systematic review concluded that task shifting offers high-quality and cost-effective HIV care to more patients than physician-centred models [9]. The results of two clinical trials confirmed these findings by showing that nurse-monitored ART was non-inferior in terms of virological suppression and retention in care [10] and that nurse-based initiation and follow-up (FUP) of ART resulted in a similar mortality rate as compared to physician-based care [11]. Cohort studies in settings with decentralized HIV care have uniformly reported favourable outcomes, including improved retention in care [12–19]. However, most studies assessed short-term clinical outcomes in pilot programmes focusing on a single district, and the generalizability of these findings is unclear. Data on the outcomes of full decentralization in regard to start and FUP of ART at the health centre (HC) level are still scarce. A recent Cochrane Review, including two cluster-randomized trials and 14 cohort studies, found moderate quality of evidence that partial decentralization (ART started by physicians at hospitals and FUP decentralized to nurse-led HCs) probably reduces attrition. For full decentralization (start and FUP of ART at the HC level), their analysis was inconclusive due to very low quality of evidence [20].

Lesotho has the third-highest HIV prevalence in the world and is particularly hit by the shortage of human resources for health [21–23]. In 2007, it was one of the first countries to decentralize the initiation and FUP of ART to nurse-led HCs on a national scale. This was facilitated by the development of national guidelines tailored to nurses who work in primary healthcare settings [24]. In a recent study of ART outcomes in rural southern Africa, we showed that among patients treated in Lesotho, only 55% were alive and in care three years after enrolment [25]. The aim of this study is to assess the effectiveness of decentralized nurse-based ART programmes by comparing three-year outcomes between patients who initiated ART at HCs and those who started treatment at hospitals in two geographically and demographically different catchment areas in rural Lesotho. By stratifying all analyses by gender, we intended to assess differences in outcomes between women and men starting ART in HCs and hospitals.

## Methods

### Study setting

The study includes data from the catchment areas of two hospitals in rural Lesotho. Seboche, located in Botha-Bothe district in northern Lesotho, has an estimated population of 55,000. It is served by one hospital and five HCs. Paray belongs to the mountainous district of Thaba-Tseka in central Lesotho, has about 77,000 inhabitants and is served by one hospital and seven HCs. According to the 2009 Demographic and Health Survey, Thaba-Tseka had an adult HIV prevalence of 20.1% and Botha-Bothe of 15.9%. Both areas are rural, and the majority of people are subsidence farmers. Compared to Seboche, Paray is more remote, with longer distances to the healthcare facilities, and its population is poorer, has a lower literacy rate and is confronted with higher levels of food insecurity and malnutrition. It was estimated that the proportion belonging to the lowest wealth quintile was 53.7% in Paray and 20.4% in Seboche, and the rate of stunting among children was 23.1 and 9.5%, respectively [26].

The two hospitals started providing ART in 2005. In 2007, in response to severe staff shortages, Lesotho published new national guidelines and started to implement a nurse-based model to decentralize and scale-up ART provision [12,27]. In the two study areas, this process was completed by mid-2008. However, laboratory examinations were not decentralized to the HC level. Blood samples are transported weekly from the HCs by motorbike to the referral hospitals. Nurses receive the laboratory results 3 to 7 days after blood was drawn. Both hospitals supervise the HCs within their catchment areas and serve as referral facilities. The hospitals are staffed with four physicians per site and provide laboratory services for basic analyses, including CD4 cell counts. Viral load testing is not available on site and not done routinely. The HCs are usually staffed with one registered nurse acting as a nurse-clinician and one nurse-assistant as well as 1–3 lay or community counsellors. The lay counsellors perform HIV counselling and testing and adherence monitoring and counselling, and they trace patients who are lost to follow-up (LTFU). The nurse-clinician and, in some cases, the nurse-assistant initiate ART and manage the clinical monitoring of patients. Table 1 summarizes the resources available for HIV care at the HCs and hospitals. There are no data on the actual travel times of patients to the facility. However, we estimate that average travel times are 2 and 1.5 hours to the Paray and Seboche hospitals, respectively. The estimated average travel time to the HCs is shorter: 1.5 and 1 hour, respectively.

**Table 1:** Resources available for ART services at the two hospitals and the 12 health centres involved in the study

	Seboche		Paray	
		
	Hospital (*n*=1)	Health centres (*n*=5)	Hospital (*n*=1)	Health centres (*n*=7)
Number of physicians	4	0	4	0
Number of nurse-clinicians per facility providing ART	3	1	2	1
Number of nurse-assistants per facility involved in ART	2	1	3	1
Number of lay counsellors per facility	5	2	7	2
Number of new adult patients enrolled in ART per facility from 1 January 2008 to 30 April 2011	873	159	832	178
Nurse-clinician/patient ratio	1/291	1/159	1/419	1/178
CD4 testing on site	Yes	No	Yes	No
Haemoglobin testing on site	Yes	2 out of 5	Yes	2 out of 7
Biochemistry on site	Yes	No	Yes	No
Viral load testing on site	No	No	No	No
TB services available	Yes	Yes	Yes	Yes
Sputum microscopy on site	Yes	No	Yes	No
X-ray on site	Yes	No	Yes	No

Both areas benefit from health projects supported by SolidarMed, a Swiss non-governmental organization (NGO). The SolidarMed ART project (SMART) has supported the implementation of the national ART programme since 2005. A more detailed description of the SMART programme can be found elsewhere [25]. The SMART programme in Lesotho is part of the International Epidemiologic Databases to Evaluate AIDS in Southern Africa (IeDEA-SA), a collaboration of ART programmes from seven countries in the region [28].

### Data collection

Individual patient data are obtained from the national ART patient sheets that are completed for all patients initiated on ART in Lesotho. Since 2005, data-managers have visited the facilities monthly to enter the paper-based data into an electronic database. Each patient starting ART in Lesotho receives a unique national identifier number, which is kept in case of transfer. It is entered into the database and allows identification of the facility at which patients initiated ART. The registered data contain information on basic demographic indicators (age and sex) as well as clinical (WHO stage, opportunistic infections, drug-related side effects, concurrent tuberculosis and pregnancy) and laboratory parameters (CD4 cell count, haemoglobin and creatinine). A data coordinator performs regular quality checks of the database, and additional checks are performed by SolidarMed staff and IeDEA-SA data managers.

### Inclusion criteria and outcomes

All patients aged 16 years or older who started ART with at least three drugs (two nucleoside reverse transcriptase inhibitors (NRTIs) and one non-nucleoside reverse transcriptase inhibitor (NNRTI)) within the two catchment areas between January 2008 and April 2011 were included. We assessed the following outcomes: retention in care, no FUP, LTFU and mortality. Retention in care was defined as alive on ART and in active FUP at database closure. No FUP was defined as no visits after the initiation of ART. LTFU was defined as not returning to the healthcare facility for six months or longer after at least one FUP visit, and not being reported dead or transferred out. Patients with no FUP after ART initiation were excluded from analyses of LTFU and mortality. Transfers of patients had to be confirmed by a national transfer letter. The analysis closure date was October 31, 2011. Patients who started ART within six months prior to the closure date could not be classified regarding LTFU and were excluded from all analyses. FUP was censored three years after initiation of ART.

### Statistical analyses

Comparisons were made between hospitals and HCs. All analyses were stratified by gender. Baseline characteristics were compared using Mann-Whitney and Pearson's chi-square tests for continuous and categorical variables, respectively. Retention in care was examined in Kaplan-Meier curves and log rank tests. We used a multivariable logistic regression model to identify individual and programmatic characteristics associated with no FUP, and we used competing risk regression to assess risk factors for LTFU or death [29]. The competing risk model accounts for the fact that the risk of death and LTFU may not be mutually exclusive [30]. Three-year retention in care at HCs versus hospitals was assessed using multivariable logistic regression. All multivariable models included the following individual and programmatic characteristics: age (16–29, 30–39 or ≥40 years), CD4 cell count (0–49, 50–99, 100–199 or ≥200 cells/µL), clinical WHO stage (I/II or III/IV), type of NNRTI (nevirapine (NVP) or efavirenz (EFV)) and catchment area (Seboche or Paray).

### Ethical considerations

This study was approved by the Ethical Committee of the Ministry of Health and Social Welfare of Lesotho. The collaboration with IeDEA-SA was approved by the Institutional Review Board of Baylor College of Medicine Children's Foundation Lesotho.

## Results

### Patient characteristics

**Figure 1:**
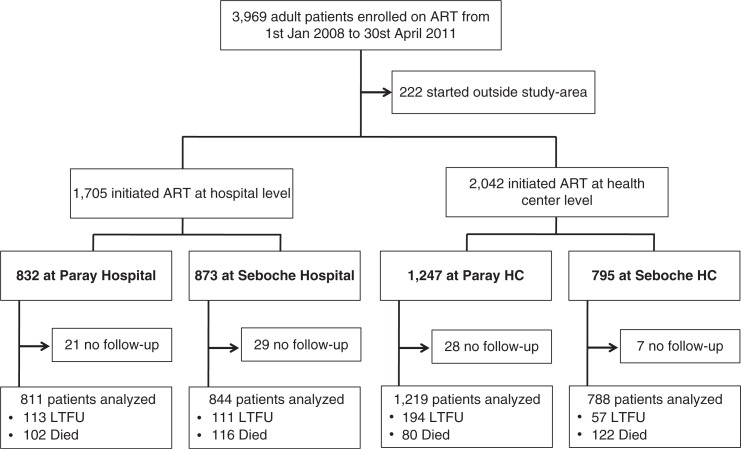
Flow chart of patients starting antiretroviral therapy (ART) in health centres (HCs) and hospitals (flow charts stratified by gender are displayed in the Supplementary file). LTFU: Lost to follow-up

Between January 2008 and April 2011, 3969 adult patients were enrolled in the SolidarMed ART programme in Lesotho. Of these, 222 (5.6%) were excluded from the analyses as they had initiated treatment elsewhere. Of the remaining 3747 patients, 2042 (54.5%) started ART at one of the 12 HCs, and the others at one of the two hospitals (Figure 1). Flow charts stratified by gender are shown in the Supplementary file. Patients in HCs had less advanced clinical disease and higher CD4 cell counts and haemoglobin levels than those in hospitals. These differences were consistent across gender (Table 2) and catchment area (Web-Table 1). Patients in hospitals were more likely to start a tenofovir-containing ART regimen than those at HCs, and, in the Paray region, they were more likely to be prescribed EFV than patients in HCs. Finally, sex and age distribution were similar in HCs and hospitals.

**Table 2:** Baseline characteristics of patients starting ART in hospitals and health centres (HCs), stratified by gender (see Web-Table 1 for stratification by catchment area)

	Female patients (*n*=2395)			Male patients (*n*=1352)			
		
	Hospital	HC	*p*	Hospital	HC	*p*	Total
Number of patients	1086	1309		619	733		3747
Median age in years (IQR)	35 (30–45)	39 (33–49)	0.021	37 (30–48)	42 (35–50)	0.017	38 (31–48)
Median CD4 cell count/µl (IQR)	183 (86–281)	223 (142–296)	<0.001	138 (60–233)	196 (102–276)	<0.001	194 (102–281)
CD4 categories (cells/µl) (%)			<0.001			<0.001	
0–49	137 (12.6)	77 (5.9)		117 (18.9)	87 (11.9)		418 (11.2)
50–99	163 (15.0)	100 (7.6)		112 (18.1)	83 (11.3)		458 (12.2)
100–199	267 (42.6)	368 (28.1)		169 (27.3)	191 (26.1)		995 (26.6)
≥ 200	486 (44.8)	730 (55.8)		197 (31.8)	344 (46.9)		1757 (46.9)
Missing	33 (3.0)	34 (2.6)		24 (3.9)	28 (3.8)		119 (3.2)
Median haemoglobin (g/dl)	11.3 (10.0–12.6)	12 (11.0–13.0)	<0.001	12.4 (10.8–14.0)	12.9 (11.5–14.7)	0.004	11.9 (10.6–13.2)
Missing haemoglobin (%)	422 (38.9)	889 (67.9)	<0.001	272 (43.9)	513 (69.9)	<0.001	2096 (55.9)
WHO stage (%)			<0.001			<0.001	
I/II	607 (55.9)	959 (73.4)		242 (39.1)	426 (58.2)		2234 (59.7)
III/IV	479 (44.1)	348 (26.6)		377 (60.9)	306 (41.8)		1510 (40.3)
NNRTI			0.003			0.044	
NVP based (%)	421 (38.8)	589 (45.0)		126 (20.4)	184 (25.1)		1320 (35.2)
EFV based (%)	658 (60.6)	416 (54.7)		488 (78.8)	547 (74.6)		2409 (64.6)
NRTI							
3TC/D4T (%)	175 (16.1)	312 (23.8)	<0.001	82 (13.3)	181 (24.6)	<0.001	749 (19.9)
3TC/AZT (%)	339 (31.2)	526 (40.2)	<0.001	156 (25.2)	179 (24.4)	0.740	1198 (31.9)
3TC/TDF (%)	567 (52.2)	469 (35.8)	<0.001	381 (61.6)	371 (50.6)	<0.001	1782 (47.6)

IQR: interquartile range; NNRTI: non-nucleoside reverse-transcriptase inhibitor; NtRTI: nucleoside analogue reverse-transcriptase inhibitor; 3TC: lamivudine; D4T: stavudine; AZT: zidovudine; TDF: tenofovir.

### Retention in care

Over 5445 person-years of FUP, overall retention in care was 66.6% (95% CI: 64.5–68.6) at three years. Disaggregated by facility type, three-year retention in care was 68.8% (95% CI: 65.7–71.6) in HCs and 64.1% (95% CI: 61.1–66.9) in hospitals.

**Figure 2:**
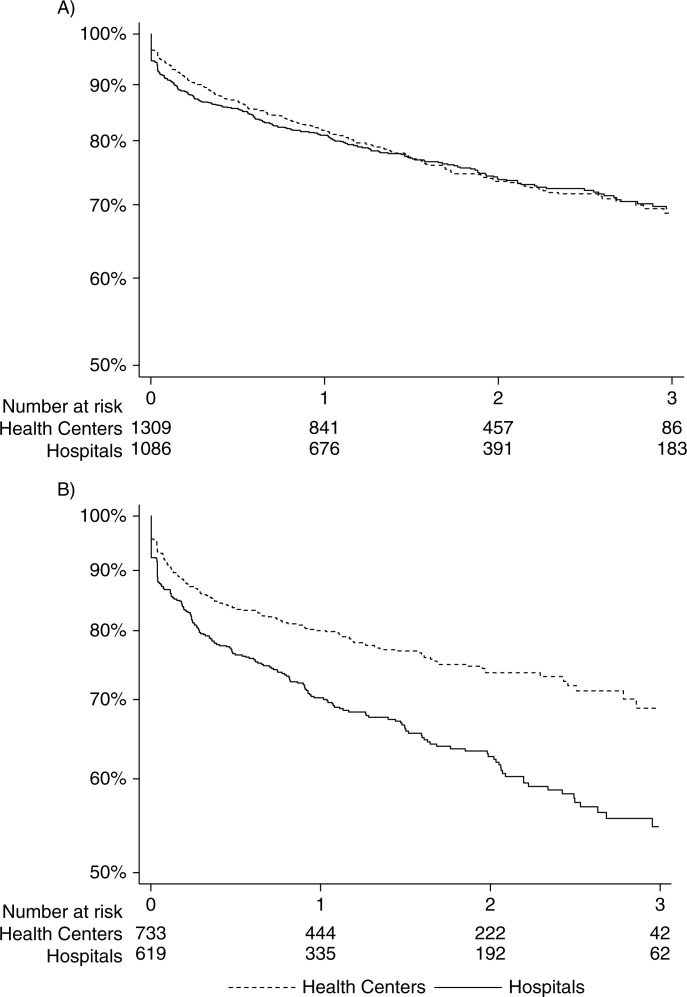
Retention in care over three years, stratified by type of facility (A: female; B: male).

Kaplan-Meier estimates for three-year retention among female patients were 69.4 and 68.7% at hospitals and HCs, respectively (*p*=0.81; Figure 2A). Among male patients, three-year retention was higher at HCs compared to hospitals: 68.8% versus 54.7% (*p*<0.001; Figure 2B). The difference in the proportion of male patients retained in care between HCs and hospitals was consistent across catchment areas (Paray: 71.1% versus 57.3% at three years, *p*=0.002; and Seboche: 65.9% versus 52.3%, *p*=0.001).

In adjusted logistic regression, men treated at HCs were significantly more likely to be retained at three years compared to those treated at hospitals (odds ratio (OR): 1.53, 95% confidence interval (CI): 1.20–1.96). There was no significant difference in retention in care at three years between women who started ART at HCs and those who initiated at hospitals (OR: 0.89, 95% CI: 0.73–1.09) (Table 3). These findings were consistent across catchment areas (retention in men (HCs versus hospitals): Paray: 1.51 (1.08–2.11), and Seboche: 1.45 (0.99–2.13); and retention in women: Paray: 0.83 (0.63–1.09), and Seboche: 0.98 (0.72–1.33)).

**Table 3:** Health centres versus hospitals: adjusted outcomes by gender (hospitals are the reference)

	Overall	Male	Female
Retention	1.10 (0.94–1.28)	1.53 (1.20–1.96)	0.89 (0.73–1.09)
No follow-up	0.62 (0.39–0.98)	0.67 (0.31–1.43)	0.60 (0.34–1.07)
Mortality	0.98 (0.80–1.19)	0.79 (0.59–1.06)	1.19 (0.91–1.57)
LTFU	0.98 (0.82–1.18)	0.68 (0.51–0.93)	1.21 (0.95–1.51)

The estimates are ORs for retention and no FUP, and sub-distribution hazard ratios from competing risk regression for mortality and LTFU. Analyses are adjusted for age, baseline CD4 cell count, clinical WHO stage, type of NNRTI and catchment area. LTFU: Lost to follow-up.

### Determinants of retention in care, by gender

Thirty-two women (2.9%) who started ART in hospitals and 22 (1.7%) who started in HCs had no FUP visit. Among men, 18 (2.9%) in the hospitals and 13 (1.8%) in the HCs had no FUP. In adjusted logistic regression, patients enrolled at HCs were less likely to have no FUP visit compared to those at hospitals (OR 0.62, 95% CI: 0.39–0.98). After stratification by gender, the point estimates remained similar in men and women (Table 3).

During the study period, 420 patients (11.5%) with at least one FUP visit died and 475 (12.9%) were LTFU. Overall, the proportion of patients who died or were LTFU was slightly lower in HCs compared to hospitals (10.1% vs. 13.5% of patients died, and 12.7% vs. 13.9% were LTFU). In adjusted analyses, male patients were less likely to be LTFU in HCs compared to hospitals (OR: 0.68, 95% CI: 0.51–0.93), but this was not true for female patients (OR: 1.21, 95% CI: 0.95–1.51). For both sexes, there was no significant difference in mortality between HCs and hospitals (Table 3).

## Discussion

We compared clinical outcomes between patients who initiated ART at HCs and hospitals in two geographically distinct rural catchment areas in Lesotho. Across gender and catchment areas, HCs enrolled patients with less advanced clinical disease and better immunological status. In line with recent publications on task shifting of ART delivery, overall retention in care was at least as high at HCs compared to hospitals [15,17–19]. However, in stratified analyses, men enrolled at HCs were more likely to be retained in care compared to those at hospitals, whereas among women, retention was similar in all settings. These results were mainly driven by the high proportion of male patients LTFU after starting ART at hospitals.

Several cohort studies have demonstrated favourable short-term outcomes in patients who were treated in decentralized ART programmes in sub-Saharan Africa [12–14,16–18] However, reports on direct comparisons of three-year retention in care between patients treated in hospitals and those in fully decentralized settings, where both ART initiation and FUP are shifted to nurses, are scarce. In a previous study from Lesotho, Cohen *et al*. showed that 77% of adults were alive and on ART two years after enrolment, but they did not specifically compare clinical outcomes between hospitals and HCs [12]. In a study of 16 HCs in Malawi, mortality and LTFU rates were lower in nurse-led HCs compared to their affiliated hospital [31]. Of note, most of the HCs in this programme only managed the FUP of patients on ART and did not initiate treatment (partial decentralization). Recently published evidence from three clinical trials confirmed these encouraging observational cohort data. In the Comprehensive International Program of Research on AIDS trial in South Africa, nurse-monitored ART was non-inferior to doctor-led monitoring in terms of virological and clinical outcomes [10]. In a cluster-randomized trial, Fairall *et al*. showed that task shifting of ART initiation and management to nurses was safe, with equivalent mortality and rates of virological suppression compared to the doctor-led model [11]. Finally, in Uganda, Jaffar and colleagues showed that for monitoring of patients who are on ART, an even higher degree of task shifting was non-inferior to more conventional approaches. They showed that patients monitored at home by trained and closely supervised lay cadres had similar outcomes in terms of mortality and viral suppression compared to patients followed at the facility [32]. However, conditions in a trial often differ from routine settings. Thus, the results of these trials might not be transferrable to routine HIV care in high-burden rural settings with scarce health resources, as is the case in Lesotho. Our study provides evidence that even in rural, resource-constrained settings with limited infrastructure and less intensive support to nurses than in clinical trials, task shifting and full decentralization seem to be a promising approach for the delivery of HIV care. We showed that when patients start ART in HCs in rural Lesotho, retention in care is at least as favourable as in hospitals, both in the short term and in the long term.

In our study, patients at HCs started ART at an earlier clinical and immunological stage than those in hospitals. This difference was consistent across gender and catchment areas. Previous studies from resource-limited settings [17,18,31,33] as well as industrialized countries [34] have reported similar findings. This may be explained by the tendency of patients who feel sick to primarily seek care at a hospital, or because severely ill patients are referred to hospitals. As a consequence, most patients with severe opportunistic diseases initiate ART while still hospitalized. Facilitating access to ART is one of the major reasons for advocating decentralization of ART delivery. Several studies described significantly lower travel costs for patients on ART at HCs as compared to hospitals [15,35]. Thus, patients who are seen at HCs might have less advanced stages of disease because of easier access to HIV care at the HC level. Overall, decentralization of ART delivery seems to be associated with earlier presentation to HIV care, which might give many patients the opportunity to start ART earlier in the course of the disease. As an increasing number of patients will have an indication to start ART in the next few years [36], decentralization of care will play a central role in the scale-up of ART. Starting ART at a very early stage of disease is endorsed by a recent study from Uganda reporting good retention and very low mortality among patients starting ART at a CD4 cell count >350 cells/µl [37].

Overall crude retention in care was higher in patients who started ART at HCs. However, after disaggregation by gender, only men had a significantly higher retention at HCs compared to hospitals, whereas in women there was a tendency towards lower retention at HCs. Several studies from cohorts in rural sub-Saharan Africa have described poorer short- and long-term outcomes among male patients [38–41]. To our knowledge, the published studies comparing long-term retention in care between HCs and hospitals did not show data disaggregated by gender. In our study, the higher retention among men treated at HCs is mainly determined by significantly lower rates of LTFU among men at HCs compared to hospitals. Although these findings are difficult to explain, our main hypothesis is that this may be employment related. It is plausible that in rural Lesotho, where most men work either as subsistence farmers or as labourers in the construction or mining industries in neighbouring South Africa, the easier accessibility of HCs may be of particular benefit to men. This is in line with the recent finding that in Lesotho, migrant workers are particularly at risk to be LTFU [42].

A strength of our study is that it was able to compare retention in care between hospitals and HCs in two different areas in rural southern Africa. In Lesotho, patients not only are followed but also have been started on ART at nurse-led HCs since 2007, which enabled us to report on relatively long-term clinical outcomes. To our knowledge, this is the first study reporting on outcomes in HCs and hospitals that is stratified by gender. In addition, the SolidarMed database records all movements of patients within the programme and keeps track of transfers out, limiting the overestimation of true losses to FUP.

This study has several limitations. Firstly, it is an observational analysis in which patients were not randomly assigned to initiating ART at either an HC or a hospital. Thus, we compared two cohorts with differences in baseline characteristics, and potential residual confounding might have affected our results. Secondly, our multivariable models did not include baseline haemoglobin level as the majority of individuals did not have a documented haemoglobin value at baseline. Baseline haemoglobin has been shown to be an independent predictor of early mortality in patients living with HIV who are starting ART [43]. Thirdly, we have no information on possible “self-transfers” who may have left one facility without a transfer letter and continued ART elsewhere. Namusobya and colleagues report from Uganda that silent transfers lead to a considerable overestimation of LTFU [37]. However, these findings may not be applicable to our study. A tracing survey conducted in 2011 in Seboche has shown that of 175 patients LTFU who were traced, only 13 (7%) had changed clinics. The majority of those LTFU were dead (33%) or had migrated to South Africa (26%) [44]. Finally, we did not have any information on the causes of death, ascertainment of death was limited and we could not rely on the systematic tracing of patients LTFU at all sites. It is known that standard mortality estimates underestimate true programme mortality as patients LTFU are at increased risk of death [45,46]. The data from the survey on those LTFU in Seboche indicate that one-third of those LTFU may be dead [44]. However, no data from a systematic tracing survey are available from Paray. In this area, mortality may be particularly underestimated, as the previously described socio-economic and geographical conditions have a negative impact on the tracing of these patients and ascertainment of outcomes is rare.

It is not clear whether the findings of our study may apply to other settings in Africa. The setting of our study is fairly unique due to the small districts and particular geographic conditions of Lesotho compared to other African countries. Moreover, the support of SolidarMed likely increased the quality of care at HCs. HCs may perform less well in settings without any support from NGOs or other implementing partners. More studies from different settings comparing full decentralization of ART to hospital-based ART are needed to examine the external validity of our findings.

## Conclusions

Task shifting and decentralization have become common practices in rural, resource-limited settings to scale up the provision of ART. Our study provides important data from Lesotho, a country with a particularly high HIV prevalence and one of the pioneers in task shifting of HIV care. This information is valuable considering the few large-scale direct comparisons of different strategies for ART delivery performed outside of South Africa. Our study indicates that rural nurse-led HCs perform at least as well as their parent hospitals: HCs had fewer early losses, and long-term retention in care was comparable to that in hospitals. Starting and following ART at the HCs appear to be beneficial for men: those treated at HCs had a higher three-year retention compared to men in hospitals, mainly because they were less likely to be LTFU in these facilities. Although this may be explained partly by the lower proportion of patients with advanced clinical disease in HCs, other reasons such as a lower number of patients per caregiver, the proximity of the treatment facility and lower transport costs might play important roles.
